# Micro-Computed-Tomography-Guided Analysis of In Vitro Structural Modifications in Two Types of 45S5 Bioactive Glass Based Scaffolds

**DOI:** 10.3390/ma10121341

**Published:** 2017-11-23

**Authors:** Fabian Westhauser, Francesca Ciraldo, Preethi Balasubramanian, Anne-Sophie Senger, Gerhard Schmidmaier, Arash Moghaddam, Aldo R. Boccaccini

**Affiliations:** 1 HTRG—Heidelberg Trauma Research Group, Center of Orthopedics, Traumatology, and Spinal Cord Injury, Heidelberg University Hospital, Schlierbacher Landstraße 200a, 69118 Heidelberg, Germany; anne-sophie.senger@med.uni-heidelberg.de (A.-S.S.); gerhard.schmidmaier@med.uni-heidelberg.de (G.S.); 2 Institute of Biomaterials, University of Erlangen-Nuremberg, Cauerstr. 6, 91058 Erlangen, Germany; francesca.elisa.ciraldo@fau.de (F.C.); preethi.balasubramanian@fau.de (P.B.); 3 ATORG—Aschaffenburg Trauma and Orthopedic Research Group, Center for Trauma Surgery, Orthopedics, and Sports Medicine, Klinikum Aschaffenburg-Alzenau, Am Hasenkopf 1, 63739 Aschaffenburg, Germany; arash.moghaddam@klinikum-ab-alz.de

**Keywords:** bioactive Glass, polyurethane foam, maritime national sponge, µCT, tissue engineering, in vitro, dissolution behavior

## Abstract

Three-dimensional 45S5 bioactive glass (BG)-based scaffolds are being investigated for bone regeneration. Besides structural properties, controlled time-dependent alteration of scaffold morphology is crucial to achieve optimal scaffold characteristics for successful bone repair. There is no in vitro evidence concerning the dependence between structural characteristics and dissolution behavior of 45S5 BG-based scaffolds of different morphology. In this study, the dissolution behavior of scaffolds fabricated by the foam replica method using polyurethane foam (Group A) and maritime sponge *Spongia Agaricina* (Group B) as sacrificial templates was analyzed by micro-computed-tomography (µCT). The scaffolds were immersed in Dulbecco’s Modified Eagle Medium for 56 days under static cell culture conditions and underwent µCT-analysis initially, and after 7, 14, and 56 days. Group A showed high porosity (91%) and trabecular structure formed by macro-pores (average diameter 692 µm ± 72 µm). Group-B-scaffolds were less porous (51%), revealing an optimal pore size distribution within the window of 110–500 µm pore size diameter, combined with superior mechanical stability. Both groups showed similar structural alteration upon immersion. Surface area and scaffold volume increased whilst density decreased, reflecting initial dissolution followed by hydroxycarbonate-apatite-layer-formation on the scaffold surfaces. In vitro- and/or in vivo-testing of cell-seeded BG-scaffolds used in this study should be performed to evaluate the BG-scaffolds’ time-dependent osteogenic properties in relation to the measured in vitro structural changes.

## 1. Introduction

Bone defect treatment remains one of the most demanding challenges in modern orthopedic surgery [1]. Since the gold standard in therapy of bone defects—autologous bone grafting—is limited due to the amount of bone to harvest, synthetic bone substitutes are the most appropriate alternative [2].

However, standard materials such as beta-tricalciumphosphate (ß-TCP) or hydroxyapatite (HA) are limited in their osteostimulative capabilities [2]. Bioactive glasses (BGs) have been shown to be a suitable alternative to calcium phosphate ceramics [3,4,5]. The discovery of 45S5 BG (45% SiO_2_-24.5% Na_2_O-24.5% CaO-6% P_2_O_5_ in wt %) by Hench et al. in the late 1960s [6] and the impressive developments in the field of BG for bone tissue engineering applications in the last 15 years have led to a wealth of information about BGs, in particular in relation to their ability to bond to bone and soft tissue [7,8]. 45S5 BG is osteoconductive and osteogenic, making it a Class A biomaterial. This glass composition develops a hydroxycarbonate apatite (HCA) layer in physiological solutions and the HCA crystals, which should form also in vivo and can connect with layers of collagen fibrils secreted by osteoblasts leading to strong bone-BG bonding [6,8,9]. The bonding occurs within days in rats and within weeks in primates and the strength of the interfacial bond formed between 45S5 BG and bone is equal to or greater than the strength of the bone itself [10]. A series of events take place at the surface of BG, including exchange of ions, condensation and repolymerization of a silanol layer, migration of Ca^2+^ and PO_4_^3−^ groups to the surface to form an amorphous calcium phosphate layer and crystallization of the CaO-P_2_O_5_ layer to form HCA crystals. In addition, in vitro studies on dissolution products of BGs have shown that relevant genes are activated when primary human osteoblasts interact with the ionic dissolution products of BG [11]. Moreover, in vivo studies have demonstrated the bone forming capability of scaffolds of different BG compositions and pore structures [12].

Recent studies have revealed that porous 45S5 BG-based scaffolds, which comprise a highly crystalline structure following the high temperature sintering of 45S5 BG [13,14], exhibit osteogenic properties in vivo [15]. Besides structural characteristics, the resorption behavior and surface alteration of scaffolds as function of time play key roles to achieve superior osteogenic effects: an “ideal” scaffold should support bone formation by controlled resorption processes and surface modifications, such as formation of an HCA layer in interaction with the host [16,17]. Both the structural characteristics as well as the resorption and surface modification processes are key features regarding the biological properties of BG-based scaffolds in vitro and in vivo [12,18].

It has previously been demonstrated that the dissolution behavior of three-dimensional (3D) BG scaffolds made from 70S30C BG can generally be observed by non-invasive micro-computed-tomography (µCT) assessment [17]. When using bioreactor systems, BG scaffolds show a strong development of HCA crystals on their surfaces in µCT analysis [17]. It has been shown that the dissolution behavior of BGs depends on the in vitro protocol and also on the structural scaffold morphology and on glass chemical composition [19]. Therefore, the release of ions to stimulate cellular growth or bone formation in later stages is directly associated with the structural characteristics of the BG-based scaffold [19,20].

It has been shown that 3D BG-based scaffolds undergo structural changes and alterations in different experimental settings over time and that these changes might be affiliated with the structural composition of the BG [14,21,22]. Whilst BG scaffolds (and other types of scaffolds) have been analyzed in different immersion settings in vitro [14,17,21,22,23], there is no in vitro evidence concerning the dependency between structural characteristics and dissolution behavior of 45S5 BG-based scaffolds of different pore structures.

In this study, we evaluated the time-dependent structural characteristics and the in vitro dissolution behavior of two different types of three-dimensional 3D-45S5 BG-based scaffolds, namely scaffolds fabricated by the foam replica method using two types of sacrificial templates: polyurethane (PU) foam with 45 ppi (pores per inch) and maritime natural sponge *Spongia Agaricina*. The scaffolds were characterized by µCT-guided evaluation following previously established protocols [24,25].

## 2. Materials and Methods

### 2.1. Bioactive Glass Scaffolds

The dissolution behavior of two different types of 3D-BG (45S5-type)-based scaffolds was analyzed by µCT as described below. Two types of sacrificial templates were used: PU foams (45 ppi) [26] and marine-sponge-inspired BG-based scaffolds (Group B) [27]. Marine sponges *Spongia agaricina* belong to the “Elephant Ears” family and have been harvested in the Indo-Pacific Ocean (Pure Sponges, Solihull, UK) in an environmentally-friendly manner, as indicated by the supplier. Bioactive glass scaffolds were produced using a melt-derived 45S5 bioactive glass (BG) powder (nominal particle size 2 µm) as starting material and by the use of the foam replica technique, according to the method developed by Chen et al. in 2006 [26].

Briefly, polyvinyl alcohol (PVA) was dissolved in deionized water at 80 °C for 1 h and the BG powder was added to the PVA-water solution at a concentration of 40 wt %. The foams were then immersed into the slurry for 10 min, and after the removal from the suspension, the slurry in excess was squeezed out. The procedure was repeated two times. After the second immersion step, the extra slurry was removed using compressed air. After drying, the foams underwent a heat treatment in order to sinter the green body and to burn out the sacrificial template. The sintering conditions were: 400 °C and 1050 °C for 1 h with a heating rate of 2 °C min^−1^ and a cooling rate of 5 °C min^−1^. After this heat treatment extensive crystallization of 45S5 BG occurs so that scaffolds exhibit a type of glass-ceramic structure [13]. The scaffolds were cut manually in cylindrical shape with nominal height of 3 mm and diameter of approximately 4 mm. The BG scaffolds were not machined (cut) after fabrication. They were obtained in cylindrical shape, which was achieved because the sacrificial foams had been cut in cylindrical shape, manually, using a 5 mm diameter punch and hammer. Five scaffolds of each group were used for the immersion experiment.

### 2.2. Immersion Method and pH Measurement

The BG-based scaffolds were stored in cryo-vials (Greiner Bio-One, Frickenhausen, Germany) containing 2.25 mL Dulbecco’s Modified Eagle Medium (DMEM), 4.5 g/L Glucose, 0.11 g/L Sodium Pyruvate, no L-Glutamine (all Thermo Fisher Scientific, Dreieich, Germany) under standard static cell-culture conditions (37 °C, 74% N_2_, 21% O_2_, and 5% CO_2_). Prior to the initial transfer of the dry BG-scaffolds into the cryo-vial, DMEM was dropped onto the scaffolds to ensure the samples were well soaked in DMEM and to reduce artifacts in µCT analysis caused by parts within the scaffolds that could remain unfilled. During µCT-scanning, the DMEM was not removed from the vial, so the scaffolds were scanned in a liquid surrounding. Medium changes were performed twice a week. The BG-based scaffolds were immersed for eight weeks (56 days). Parallel to µCT-assessment, the medium was collected and frozen at −20 °C prior to pH measurement with a benchtop pH meter (PB-11-P10.1M; Sartorius, Göttingen, Germany).

### 2.3. µCT Acquisition, Dataset Reconstruction

µCT-scans were performed with a SkyScan 1076 Hasitom (Bruker microCT, Kontich, Belgium) µCT using established acquisition protocols, following recent recommendations [15,28,29,30]. Before acquisition, flat-field correction and alignment were checked and corrected according to the manufacturer’s instructions to ensure reproductive scanning conditions. The acquisition details were: tube current 200 µA, integration time 450 ms, voltage 50 kVp, pixel size 9 µm, 0.5 mm Al filter. Scans were made starting from the first day of immersion in DMEM (T0), followed by a scan after seven days of incubation (T1), after 14 days (T2), concluded by a final scan after 56 days of immersion (T3). The samples were stored in cryo-vials during the scanning procedure and were kept wet. NRecon (Version 1.6.9.8; Bruker microCT, Kontich, Belgium) was used for 3D-reconstruction. A beam hardening correction of 10 and ring artifacts reduction of 6 were applied for all samples. Misalignment compensation was evaluated individually. During reconstruction, Hounsfield unit (HU) calibration was performed. Datasets were saved as tiff-files.

### 2.4. µCT-Data Evaluation

The reconstructed files were evaluated using Heidelberg-µCT-Analyzer for structural analyses [24]. The algorithm calculates a threshold by automated analysis of the scaffold using a combination of Outsu’s method and fuzzy clustering. The obtained threshold is applied to the scaffold for segmentation, binarizing the volume of interest (VOI) into the actual scaffold structure (“positive pixels”) and the “non-scaffold” structures, such as empty spaces (pores, in this case) inside the scaffold (“negative pixels”). From the segmented dataset, several structural parameters are calculated. In this study, the scaffold’s most important 3D-features and their change during immersion were analyzed by evaluation of total volume (TV), scaffold volume (SV), surface area (SA), and the density of the scaffold structure (TMD_SV). Furthermore, the initial structural characteristics including TV, SV, and SA as well as porosity (P), pore number (PN), rPN (PN for pores with a diameter between 110 and 500 µm), and mean pore size (PS), representing the average pore diameter were analyzed. In short, TV, SV, SA, and TMD_SV were calculated from the segmented scaffold (“positive pixels”). TMD_SV was calculated by referring the grey-values of each VOI inside SV to the global threshold for SV. PN, rPN, and PS were calculated from the extracted “empty” spaces (“negative pixels”) inside the scaffold structure.

**Figure 1:**
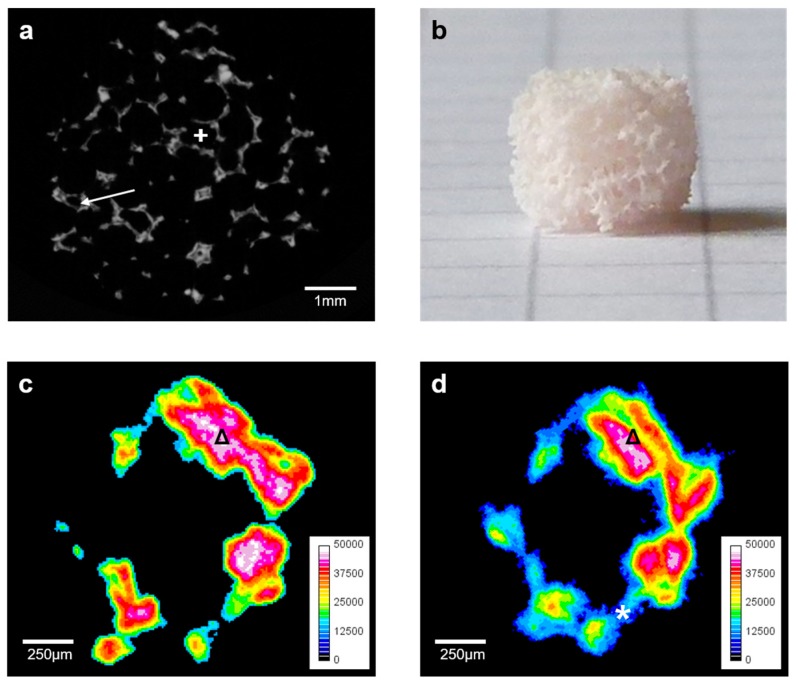
Characterization of Group A scaffolds: representative 2D-µCT-image (**a**); a photograph of the scaffold (edge length of the squares in the background: 5 mm) (**b**); 2D density color-coded µCT-slices (calibration bar in Hounsfield unit (HU)) of a defined scaffold region at T0 (**c**) and after 56 days in immersion (**d**). The 2D-micro-computed-tomography (µCT)-slide shows the typical trabecular structure of the BG-based scaffold (**a**), with some smaller pores surrounded by the glass (→) and the dominating larger pores (+). The high interconnectivity can be anticipated optically. Over time, a decrease of the dense scaffold structure was detectable (Δ), however the decrease was followed by the formation of lower density material on the scaffolds boundaries (*). This could explain the overall decrease of the density and the overall increase of total volume (TV) and scaffold volume (SV) over time.

**Figure 2:**
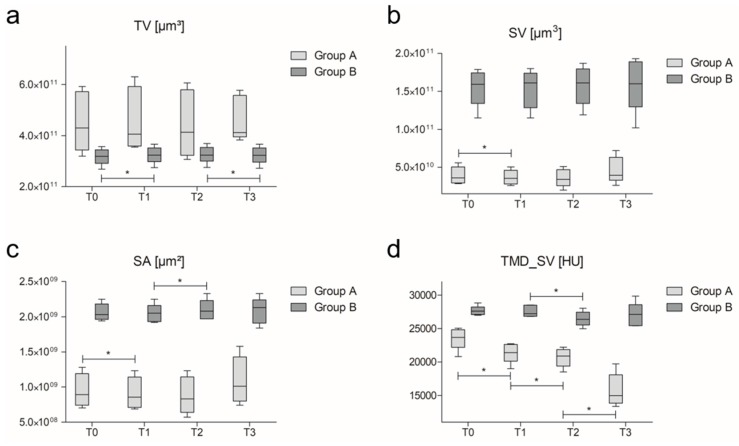
Data for the µCT-characteristics for TV (**a**) SV (**b**), SA (**c**), and TMD_SV (**d**) of the analyzed scaffolds (groups A and B) over time in Dulbecco’s Modified Eagle Medium (DMEM) (T0: initial scan at the beginning of the immersion period, followed by scans after seven (T1), 14 (T2), and 56 (T3) days in immersion), shown as boxplots (mean values with SEM and range). Significant changes over time are marked by (*).

Color-coding according to the respective density of the scaffolds and image scaling was performed with ImageJ (Version 1.51j8; US National Institutes of Health, Bethesda, MD, USA) by normalizing the density gradient for the analyzed datasets to a range of 0–50,000 HU followed by conversion of the grey-scaled image file to a 16 colors image. After adding a reference color scale, the density was optically and qualitatively analyzed as shown in Figure 1c,d and Figure 2c,d. The analysis of HCA formation on the surface was conducted for T3, using established protocols with some modifications [23,31]. In short, reconstructed datasets were opened in CTAn (Version 1.13.2.1+; Bruker microCT, Kontich, Belgium). HCA-threshold was defined by manually fitting a region of interest (ROI) on the scaffold’s surface. The threshold from the ROI was applied to the whole dataset to calculate absolute HCA-volume. Absolute HCA-volume was then divided by the scaffold volume (SV) to assess percentage amount of HCA compared to the volume of the whole scaffold (SV).

### 2.5. Statistical Methods

SPSS Version 22 (IBM Corporation, Armonk, NY, USA) was used for statistical analysis. Graphs were created using GraphPad Prism Version 5.01 (GraphPad Software, La Jolla, CA, USA). Results were analyzed with the Mann-Whitney U Test for independent samples. Paired samples were analyzed by Wilcoxon signed-rank test. Results were described as statistically significant for *p* < 0.05. Data are shown as rounded mean values followed by the standard deviation of the mean in brackets.

## 3. Results

### 3.1. Macroscopic and Haptic Findings

**Figure 3:**
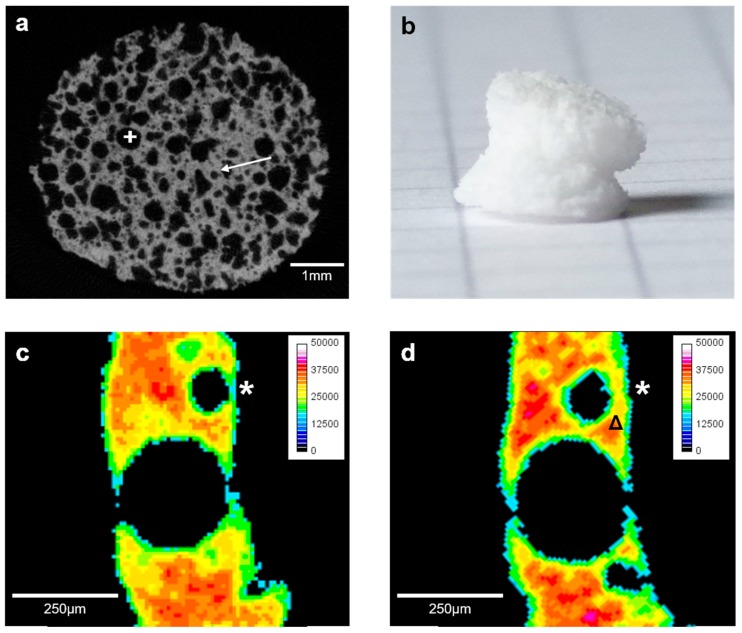
Characterization of Group B scaffolds: representative 2D-µCT-image (**a**); a photograph of the scaffold (edge length of the squares in the background: 5 mm) (**b**); 2D density color-coded µCT-slices (calibration bar in HU) of a defined scaffold region at T0 (**c**) and after 56 days in immersion (**d**). The 2D-µCT-slide shows the typical structure of the bioactive glass (BG)-based scaffold (**a**), with medium sized pores (+) surrounded by smaller pores (→). During immersion, the boundaries of the scaffold became thicker, most likely because of hydroxycarbonate apatite (HCA) formation as a result of BG dissolution (*). The outer shell of the scaffold remained with a lower density, however, the parts underneath (Δ) showed higher density.

The A-Group scaffolds were more fragile compared to the B-Group scaffolds when handling them with forceps. Macroscopically, the A-scaffolds showed a macro-porous character (Figure 1b), and the B-scaffolds appeared to be more robust and less porous (Figure 3b). All scaffolds were cut manually in cylindrical shape and had an approximate average weight of 0.08 g (Figure 1b and Figure 3b). Due to the small size and the difficult handling of the brittle samples, differences in total volume (TV) were significant (Table 1), distinctive for the correlation between structural and mechanical characteristics.

### 3.2. General Aspects of µCT-Evaluation

Scaffolds of the A-group showed a trabecular character, mostly containing macropores which is typical for scaffolds made from PU foams [26]. The trabecular structure of the scaffolds indicated high pore interconnectivity (Figure 1a).

Scaffolds of the B-group were stronger and better to handle. Pores have a smaller size than in group A scaffolds, but single macro-pores are present as well. The pore interconnectivity was lower than in group A scaffolds (Figure 3a).

Quantitative µCT-evaluation was performed to analyze the structural modifications of the scaffolds during the immersion period. Table 1 summarizes the initial scaffolds characteristics, data representing the structural changes of the scaffold over time are shown in Figure 2.

### 3.3. Quantitative µCT-Evaluation of the Initial Characteristics

The T0-data of group A and B were compared and results are shown in Table 1. TV of the A-group scaffolds is significantly (42%) larger compared to the TV in scaffolds of the B-group. The analyzing software defines the outer shape of the scaffold and calculates the whole volume in the interior of the defined pattern which is referred as TV [24]. The difficulties in cutting the scaffolds mentioned above impact their measurement. While the B-groups scaffolds kept their structural integrity during the cutting process, the A-scaffolds tend to break along the boundaries. Therefore, the scaffolds of the A-group were cut into lager dimensions than the anticipated size to avoid a strong impact of the loss of material caused by the cutting process. This resulted in larger TV for scaffolds of the A-group.

SV represents the scaffold volume only. This means, that the software uses the pattern defined for TV, but only counts the “solid” parts of the scaffold as SV, or the dense parts of the scaffold structure, respectively. Consequently, the pores and unfilled spaces inside the scaffolds remain uncounted [24]. Our results demonstrate that the B-scaffolds have an almost 75% higher SV compared to the A-group, and the differences are significant (Figure 1a and Figure 3a).

SA, quantifying the scaffolds’ surface area, depends on scaffold volume, its porosity, the number of pores, and pore size. Usually, the scaffolds’ volume has a slightly stronger impact on SA compared to the pore characteristics [24]. However, in this case, parameters concerning porosity, as shown below, differed significantly, followed by a major impact on surface specification. SA in B-group was a significant 54% larger compared to SA in the A-scaffolds.

A different behavior of scaffolds during immersion as function of time caused by different TV values is possible. To assess if there is a chance for bias and to compensate the differences between the different TV caused by cutting, SV and SA were normalized to TV (SV/TV and SA/TV, respectively). The normalized scaffold volume (nSV) was with a value of 0.49 significantly higher in B compared to A scaffolds (0.09, *p* = 0.008). The normalized surface area (nSA) was with a value of 0.65/µm^−2^, significantly larger in B-group compared to A-group (0.21/µm^−2^, *p* = 0.008). The glass parts as well as the surface are therefore larger for B samples when normalizing to the different TV, making biasing less likely.

P was a significant 77% higher in the A-group compared to B-group-scaffolds. PN was the opposite: B-scaffolds have significantly more pores (75%) compared to the A-group. However, the relative number of pores within the window of 110–500 µm, represented by rPN was with 60% in the B-group significantly higher than the 28% in the A-group. In general, A-scaffolds showed a macroporous character, almost 69% of the pores had a diameter of more than 510 µm. There were only 4% of pores with ≤110 µm in diameter detected in A-group scaffolds (Figure 1a). In the B-group, the major part of the pores, 60%, was within the rPN-range. There were 37% of pores with ≤110 µm and a smaller number of macro-pores (3%) in the B-scaffolds (Figure 3a).

The average pore diameter, which is represented by PS, was significantly larger in the A-group compared to the B-group, matching the results for rPN.

### 3.4. Quantitative µCT-Evaluation over Time

For the analysis of the scaffold changes over time, we focused on the evaluation of volume, surface, and density-parameters because pore-associated changes are mostly linked to bone ingrowth [22]. These results are shown in Figure 2. Furthermore, the absolute and percentage amount of HCA formed after 56 days of immersion was calculated.

TV increased significantly (*p* = 0.043) from T0 to T1 in B-scaffolds. TV in A-scaffolds increased as well from T0 to T1, but the changes were not significant. SV decreased in the same period, even significantly (*p* = 0.043) in A-scaffolds. Comparing T1 to T2, TV showed almost no change in the B-group, and a non-significant decline in the A-group. From T2 to T3, TV showed a significant decline in B-scaffolds (*p* = 0.043), but a non-significant increase in A-samples.

SV showed an initial decrease in both groups, with a significant change in scaffolds of group A (*p* = 0.043). The decrease continued in group A; but changed in B-scaffolds toward a non-significant increase. From T2 to T3, SV increased slightly in both groups. Compared to T0, SV was lager in T3, thus has increased during the 56 days of immersion in DMEM (Table 2).

The surface area decreased from T0 to T1 significantly in the A-group (*p* = 0.043) and non-significantly in the B-group. The decrease continues in A-scaffolds from T1 to T2. In the B-group, a significant (*p* = 0.043) increase of SA was detectable within the same time period. From T2 to T3, there was non-significant increase of SA in scaffolds of group A (almost 20%) whilst scaffolds of group B remained almost unchanged.

The density of the scaffolds decreased significantly (*p* = 0.043) from T0 to T1 in the A-group and only slightly in the B-group. The decrease continues from T1 to T2 significantly in both groups (*p* = 0.043 for both), however, the absolute change in the A-group (−3.47%) was lower compared to the alterations from T0 to T1. Whilst there was a non-significant increase from T2 to T3 in the B-group, the A-group again decreased significantly (*p* = 0.043). Overall, the changes in density are indicative of dissolution of the glass structure.

Comparing the initial characteristics of the scaffolds to those after 56 days of incubation, TV, SV, and SA increased in all groups (Table 2). After normalization, the changes revealed the same tendencies, except for nSA, which slightly decreased in B. The increase of TV in scaffolds of group B over the incubation period was significant, as well as the decrease of TMD_SV in scaffolds of the A-group.

The percentage HCA formation after 56 days of immersion was 5.72% (±1.88%) for the A-scaffolds and 6.61% (±1.70%) for the B-scaffolds. Absolute HCA-volume was significantly higher in B-scaffolds (1.01 × 10^10^ µm^3^; ±0.40 × 10^10^ µm^3^) compared to A-scaffolds (0.27 × 10^10^ µm^3^; ±0.08 × 10^10^ µm^3^; *p* = 0.008).

### 3.5. pH Changes over Time

**Figure 4:**
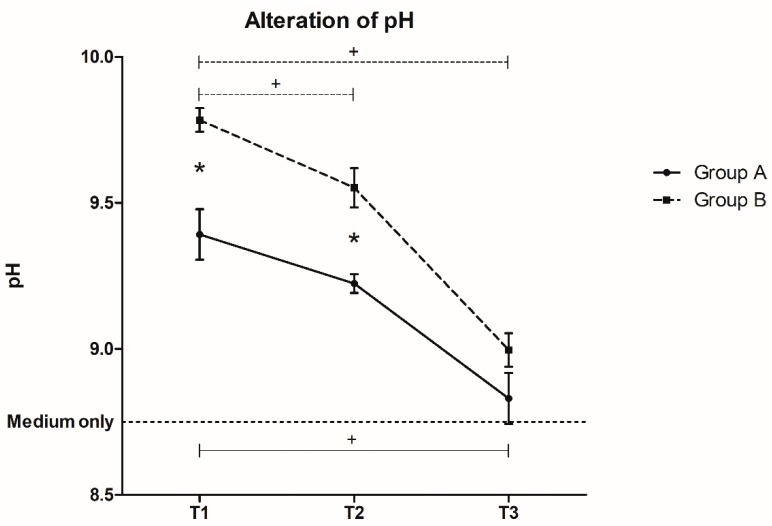
Variation of pH during immersion in DMEM. DMEM without contact to scaffolds was taken as a reference (“Medium only,” dashed line, pH = 8.75). Immersion duration is plotted on the *x*-axis according to the scan time points (T1–T3). Significant differences between group A-scaffolds and group B-scaffolds are marked with (*), significant differences in pH between two immersion time points in one scaffold group are indicated with a bracket in combination with a (+).

pH changes were analyzed parallel to the µCT scans. The pH of DMEM without contact to scaffolds was used as reference (Figure 4).

After the first week, average pH of the medium containing the A-scaffolds was 9.32 (±0.19). For the B-scaffolds, pH was significantly higher with 9.73 (±0.09; *p* = 0.016). The pH remained higher in the B-group over time, even significantly at T1 (*p* = 0.008). The pH decreased continuously towards the actual pH of DMEM over the whole immersion period from T1 to T3 in both groups (A: *p* = 0.042; B: *p* = 0.042). The pH remained at T3 non- significantly higher in both groups (A-group: 8.83 ± 0.19; *p* = 0.625; B-group: 8.99 ± 0.12; *p* = 0.063) compared to DMEM without contact to scaffolds.

## 4. Discussion

In this study, we analyzed for the first time the structure and the dissolution characteristics of 3D BG-based scaffolds manufactured by using different templates with non-invasive and standardized µCT-guided evaluation. The scaffolds were produced from 45S5 BG by the foam replica method [26] using two different sacrificial templates, namely PU foams or maritime sponges [27]. From published studies, it is well known that the internal structure of scaffolds or the resorption kinetics and the alteration and changes of scaffold surfaces are crucial for successful scaffold osteointegration and to stimulate bone growth [15,16,32].

Positive aspects of this study are the use of established protocols for both the physiological environment and the µCT-analysis to evaluate both the structural characteristics and the dissolution behavior of the scaffolds [17,24]. A limitation of the study is the lack of evaluation of the osteogenic properties of the scaffolds, for example by seeding cells or using adequate in vivo models, which remains the task of future work.

In the analysis of the initial structure of the scaffolds, the volume of both scaffold types differed significantly. Whilst TV was significantly higher in the A-group, SV was significantly larger in the B-group. In combination with the results obtained from P, the B-group scaffolds are confirmed to have a higher solid content and lower “free space” inside in comparison to A-group scaffolds. When µCT-scans are analyzed, these findings are confirmed (Figure 1 and Figure 3). It has been previously shown that the compressive strength of maritime foam derived scaffolds (comparable to group B) is superior compared to scaffolds derived from PU foam templates (comparable to group A) [27].

Porosity is one of the key features characterizing bone substitutes. 3D BG-based scaffolds (group A) investigated in vivo have shown a high osteogenic potential with a porosity of around 90% [15]. In that study, the used in vivo model, focused on ectopic, subcutaneous implantation of 3D scaffolds, seeded with human mesenchymal stem cells, confirmed the basic osteogenic properties of scaffolds. Using the described model [25], a high porosity seems to improve the osteogenic features. However, the model cannot be used to evaluate the mechanical properties of bone substitutes, because of the lack of realistic orthotopic bone-like conditions. Therefore, mechanical stability or strength does not play a role in the described model [33,34]. When using scaffolds as filling material in bone defects, the mechanical properties become important [33,35]. Furthermore, previous work indicates that porosity is of crucial importance in orthotopic models as well, making the architecture of the scaffolds a relevant factor regarding the scaffold’s biological features [12,36,37]. It is therefore of interest to evaluate the in vivo osteoinductivity of the “low-porosity” B-group-scaffolds. If bone formation would remain the same compared to scaffolds with a porosity of more than 90%, a way to improve the mechanical stability of BG-based scaffolds could be found by using maritime sponge template. Furthermore, the B-scaffolds have significantly higher amount of glass (solid material) compared to the A-group scaffolds. The availability of a solid surface is crucial for cell attachment and therefore a key factor in osteoinduction. Probably, the effect of porosity itself is overestimated, and research should be primarily focused on optimizing scaffold surfaces. However, the analysis of changes in porosity over time is of interest in the evaluation of cell-seeded bone substitutes (quantification of bone ingrowth), which may behave differently to the cell-free scaffolds investigated in this study [15,38].

The B-group scaffolds have been analyzed in terms of structural characteristics before. For example, Boccardi et al. reported a porosity of 0.68 [27]. Compared to the present results, the porosity was anticipated higher. In the previous study, µCT-analysis was also used to evaluate porosity, however the spatial resolution of the obtained images was 5.15 µm/pixel and not 9 µm as applied in this study. This means that pores with a diameter between 5.15 µm and 9 µm were analyzed in the mentioned study, but not in the present case, which can explain the small difference in porosity measured in both cases.

The relatively low resolution of the µCT system used in this study is the main limitation for the use of this technique for the detailed analysis of changes in scaffolds over time. For example, HCA-formation on the scaffolds’ surfaces can be detected in an earlier stage using either µCT systems that provide a more detailed resolution or other approaches such as electron microscopy or synchrotron-based imaging techniques [23]. However, the group of Yue et al. demonstrated that the changes in the structural aspects of scaffolds can be anticipated by µCT using a comparable resolution in a very reliable manner [17]. The µCT methodology differed between the cited study and this study in terms of the normalization methods: whilst the group of Yue et al. assumed the X-ray attenuation of parts of the scaffold remaining the same over time and can therefore be used for normalization, we referred to water and air as constant references following recent recommendations [29,30]. We then applied the X-ray attenuation-values of the references to the BG structures to determine the changes within the immersed scaffold over time. By non-destructive analysis of one and the same scaffold over time, changes can be evaluated directly providing improved follow-up conditions compared to other techniques that require the destruction of the respective specimens [17,23,31].

In literature, an optimal pore size of 110–500 µm has been defined as one of the key markers for strong osteoinduction and is analyzed as rPN in this study [15,32,34]. rPN in the B-group is significantly higher compared to the A-group. This could cause better osteoinductive properties. However, as discussed above, P and PS are significantly lower in B-scaffolds. Indeed, the actual osteoinductive properties can only be evaluated by using either cell-seeded scaffolds in vitro or in vivo [15,16,25,39].

Over the immersion period of 56 days, a significant decrease of the average density within the A-group was detected, in combination with a non-significant increase in TV, SV, and nSV. The B-group showed the same characteristics, with a significant increase of TV. These changes may be explained by HCA-formation on the surface which is expected to occur on these BG-scaffolds during incubation in medium [6,17,40]. The assumption is also supported by the pH-patterns observed: a strong initial pH increase was followed by a constant decrease over time. After 56 days of immersion, the DMEM containing the group A-BG almost reached the pH level of the DMEM without contact to the scaffolds (Figure 4). BG structures are converted along with ongoing HCA formation, resulting in less pH-alteration which has been demonstrated before [41,42,43]. This could qualitatively be confirmed by the thickening of the glass structure detected in µCT over time (Figure 1d and Figure 3d) in both groups and by calculation of the amount of HCA-formation on the scaffold surfaces. However, the newly formed HCA material appeared to be less dense than the actual scaffold structures, giving a reliable explanation for the combination of increase in volume and surface area with a decrease in density. Compared to other studies, the amount of HCA formation was with 5.72% for the A-scaffolds and 6.61% for the B-scaffolds relatively low in this case [17,23]. However, there is a positive correlation of the amount of BG and the quantity of HCA formed which is supported by the findings within this static experimental setting: significantly higher HCA formation was detected within scaffolds of the B-group. The use of bioreactor systems seems to be much more effective than immersion in static culture, however, there is no quantitative data about the in vivo dissolution behavior and HCA formation for this type of scaffolds [44]. It therefore still remains unknown which model actually comes closer to the in vivo situation, making further in vivo testing necessary [45].

The differences regarding the increase of TV in scaffolds of group B may be linked to both, the significantly larger surface area and the significantly larger SV: in these scaffolds, there is more material (BG) that can be converted to HCA followed by a stronger rate of conversion. By normalization of SV to take the larger TV of A-scaffolds into consideration, SV still remains higher in group B-scaffolds, making the results transferable, even when TV changes. TV decreased significantly from T0 to T1 in scaffolds of group A, representing a faster dissolution of the A-scaffolds compared to the B-scaffolds. In literature, there is evidence for an initial burst dissolution of similar scaffolds, especially when using bioreactors, confirming the present results [17].

SV decreased from T0 to T1 in both groups, even significantly in the A-group. The reduction of SV correlates with the dissolution of the glass: in combination with a continuous decrease in density, the dense parts of the scaffold dissolve initiating the formation of material with lower density on the surface of the scaffold (Figure 1c,d and Figure 3c,d). The dissolution of the scaffold surface is stronger during the early phase of immersion and is followed by HCA-formation on the scaffolds surface causing an overall increase in SV over time—confirming results reported in literature [17]. Over time in immersion, the newly formed layers on the surface of the scaffolds become denser over time, as indicated by µCT-analysis (Figure 3d). Furthermore, the newly formed material on the scaffold surfaces shows a rough morphological character that causes a slight increase in SA (Figure 1d and Figure 3d).

A possible explanation for the comparably minor changes over time could be the use of the specific static experimental model: static cell culture conditions without a continuous flow in the tube are associated with a slower degradation processes. It should be therefore pointed out that under more relevant physiological conditions in either a bioreactor system in vitro or in vivo, the resorption rates could differ significantly from the findings in this study [46,47].

## 5. Conclusions

The fabrication of 3D bioactive glass-based scaffolds of different pore structures is required to determine the best possible scaffold architecture leading to high osseointegration and osteoconductive potential. In terms of structural properties, new ways of production could be accompanied by better mechanical properties or superior structural features. The specific structural characteristics of scaffolds are directly related to their osteogenic properties. The tested 45S5 BG-based scaffolds exhibiting two distinct pore architectures showed similar structural changes over time: whilst volume and surface increased, density decreased over time. This behavior reflects changes on and directly underneath the surface of the BG, for example by initial dissolution followed by formation of a HCA-layer on the surface. Furthermore, the impact of structural changes seems to be related to the morphology of the scaffolds: with a lower amount of solid material, the degradation increases while with increasing BG amount (less porosity) the total volume increases. However, in vitro-testing and/or in vivo-testing of cell-seeded BG-based scaffolds used in this study should be performed to evaluate their osteogenic properties and how they relate to their different pore structure. From their favorable structural integrity and porosity, marine sponge–derived scaffolds seem to be a promising alternative to standard scaffolds obtained by using PU foams.

## Figures and Tables

**Table 1:** Data for the µCT-characteristics of the analyzed scaffolds (groups A and B) for T0, mean values with standard deviation of the mean in brackets. TV: total volume, SV: scaffold volume, SA: surface area, P: porosity, PN: pore number, rPN: relevant pore number, PS: mean pore size). Δ% represents the percentage difference comparing A- and B-groups. *p* shows the *p*-values comparing A- and B-groups; significant differences are distinguished by (*).

µCT-Characteristics	TV [10^11^ µm³]	SV [10^11^ µm^3^]	SA [10^9^ µm^2^]	P [1/µm]	PN	rPN	PS [µm]
A	4.52 (1.17))	0.39 (0.14)	0.95 (0.34)	0.91 (0.01)	7306 (1531)	2013 (210)	692 (72)
B	3.18 (0.32)	1.55 (0.25)	2.06 (0.12)	0.51 (0.03)	29,383 (2332)	17,618 (2724)	189 (15)
Δ%	−42.09	74.71	53.91	−77.44	75.14	88.57	−266.59
*p*	0.032 *	0.008 *	0.008 *	0.008 *	0.008 *	0.008 *	0.008 *

**Table 2:** Data showing the µCT characteristics of the analyzed scaffolds (groups A and B) comparing changes during the total incubation time (T0 vs. T3). Mean values with standard deviation of the mean in brackets. Δ% = 1 − (T0/T3) represents the percentage difference comparing two time points: Δ% [%]t = T0 vs. T3. *p* shows the *p*-values comparing T0 with T3. Significant differences are distinguished by (*).

		TV [10^11^ µm³]	SV [10^11^ µm^3^]	nSV	SA [10^9^ µm^2^]	nSA [/µm^−2^]	TMD_SV [HU]
T0	A	4.52 (1.17)	0.39 (0.14)	0.09 (0.01)	0.95 (0.24)	0.21 (0.01)	23,550 (1644)
	B	3.18 (0.32)	1.55 (0.25)	0.49 (0.03)	2.06 (0.12)	0.65 (0.04)	27,652 (715)
T3	A	4.63 (0.88)	0.46 (0.16)	0.10 (0.03)	1.10 (0.34)	0.24 (0.05)	15,764 (2477)
	B	3.24 (0.34)	1.60 (0.36)	0.49 (0.07)	2.09 (0.18)	0.65 (0.02)	27,023 (1814)
Δ% [%]t	A	2.30	15.26	12.73	13.14	10.45	−49.39
	B	1.70	2.71	0.47	1.10	−0.82	−2.33
*p*	A	0.893	0.225	0.409	0.225	0.786	0.043*
	B	0.043*	0.345	0.892	0.686	1.000	0.225
